# The "Artistic Temperament": The Real Artist's Temperament Analysed

**Published:** 1916-05-20

**Authors:** 


### THE EDITOR'S LETTER-BOX.

WE have received a communication on the Artistic Temperament which would occupy quite three pages, and is therefore too long for publication. All we can find space for is the latter half of it, which attempts to supply an analysis of the real artist's tempera ment and to inquire where the great difference between the artist and the artistic temperament lies.

### THE "ARTISTIC TEMPERAMENT." THE REAL ARTIST'S TEMPERAMENT ANALYSED.

*To the Editor of* THE HOSPITAL.

Sir,—As one who has habitually been accused of possessing—or should I say suffering from?—the artistic temperament, I read the article on this subject last week with keen interest.

I will make a brief attempt to supply an analysis of the real artist's temperament, the omission of which disabled your contributor's article. For the question of questions is how far artistic genius (our sole concern here) is itself a morbid symptom, while never forgetting that "a man with more brains than his fellows will necessarily seem as mad to them as he who has less." In order to answer this inquiry we must first decide what is normal. The marks of this very loosely defined diathesis in some sort being arrived at, the next point will be to ask: How much beyond the normal selfishness ought one to stand from a genius for the sake of his gifts to humanity; and how may we distinguish him from those who seem to have his vices merely? Does the artist type possess more than the average defects, as well as more than the average of a special kind of virtue? If so, since the type has proved to be as indispensable to society as the two sexes are to its survival, to complain of its defects is like complaining that grass is often wet, and sunshine often fatal. But, with shoes and sunshades, we waste no time in merely calling them names. We know that grass and damp are inseparable. How if the artist and unscrupulous selfishness be equally so? Let us see—and, if so, certainly provide ourselves with such damp-proof protection as is reasonable.

There are two chief marks of the born artist. The first is the selfishness with which he will sacrifice his own health, or anyone else's, his own life, or anyone else's, if he believes that by so doing he can compose, or paint, or chisel with deeper understanding, and create subtler harmonies of sound and thought, of colour relations, of mass and line. As the typical mother is *capable de tout* for the sake of her child, so the born artist is *capable de tout* for the sake of his offspring. Balzac writing eighteen hours a day during most of his life for the sake of his *comédie*; George Sand gobbling up one man after another for the sake of her novels; Shakespeare anatomising the very soul of the Dark Lady for his sonnets, are the merest gleaning of classical examples to prove the inveterate selfishness of the artist in sacrificing either his own health or the happiness of others for the sake of the end he has in view—a selfishness, remember, which, were it not at bottom an impersonal one, whose fruits have been religiously honoured because it adds a fresh inheritance to the race, would revolt us as the symptoms of amorous infatuation or the pains of childbirth would revolt us could children of the flesh or of the spirit be born in any other way. Like the mother, the artist in sacrificing others is sometimes sacrificed himself: in both cases to the creative purpose which masters them. But, for all this, can it be denied that the male artist is proverbially a bad husband, and the female artist a bad mother and a bad wife?

The second mark of the born artist is his laziness, by which I mean the well-observed fact that he will fight his way through incredible privation and discouragement rather than work at anything but his art. When the popular alternative between starvation as an artist and an honest living at a job he does not like is offered to him, he will unhesitatingly choose starvation, eked out (which is the real curse of poverty) wherever possible by such sponging or open assistance as his scandalised relations may afford. By these two chief marks is the artist's temperament distinguished. Add to these a large dose of average human nature, and you have him at his proper level.

If I venture to be dogmatic here it is because your contributor's curious definition of work as "doing that which is distasteful" shows that he is not himself an artist. Work is simply the expenditure of energy; and the expression of personality in production, with the healthy joy associated with it, is precisely what differentiates an artist's from the opposite extreme, called by contrast impersonal, mechanical drudgery. The arts have their drudgery, it is true; just as all men in the work that they enjoy, are artists; for, as Morris knew, art is simply the expression of pleasure in work. It is only our economic conditions which have made such a ghastly definition as your contributor's possible.

Where, then, is the "great difference" between the artist's and the artistic temperament, which your writer so hastily assumes? The difference between the dilettante and the artist is the difference between consumer and producer merely, though they shade off into one another here as everywhere else. Is there any temperamental difference between them? I venture to suggest that there is none whatever. The men, indeed, are different, but the temperaments of the twain are the same. We may distinguish between the two *men*, however, by the specific chance endowment of energy, with all its corollaries, in one of them. It is the presence or absence (with all its effects) of this alone which distinguishes the one man from the other. Theinternal combustion engine is one type of many forms, though the degree of compression in any two examples even of the same form may vary enormously. It is as much and as little true to claim that a hunchback has a different temperament from that of normal men as to attempt to divide the temperaments of the artist and the artistic person. The practical difference is immense, but it is not a temperamental one.

In any case, whether the preceding analysis of the artist's temperament be right or wrong, if your contributor will be so good as to set my picture between his own delineation of a variant from it on the one hand, and the still empty canvas of the normal type of average human being on the other, he and his readers will have a standard by which to measure the degree of difference between them; and it is the particular degree that becomes the difference which we are most concerned to know. For the practical question is, by such comparison, to discover now (since posterity's decision always comes too late) who is the genius and who is the waster—since, in face of the manifest resemblances between the two, we do not at present know where we stand, nor whom to tolerate and whom to suppress.

If I have carried him with me thus far, perhaps your contributor will go on to tell us to what extent the artist's temperament is itself a morbid symptom? If he can and will do this his preliminary step must be to lay down what is to be regarded as the standard of sanity, unselfishness, and health. As a reward for our pains, with this standard in hand we should then be in a position to attack that most elusive and alluring of questions, the nature of genius. The very word at present reeks of fog. Only by an understanding of the humdrum and the normal can the limelight of science be flashed upon it, and incidentally Psychology be rescued from her position as the Cinderella of the Sciences, where, covered with dirt, she now lies amid the dust and ashes of the unreal and remote studies to which her pretentious and purse-proud sisters, Madame von Psychiatry and Frau Research, have scornfully and, in the end, as we now see, suicidally condemned her.

I am, Sir, etc., OLE BOTHER.

